# Supplementary material for: A Critical View of the Application of the APEX Software (Aqueous Photochemistry of Environmentally-Occurring Xenobiotics) to Predict Photoreaction Kinetics in Surface Freshwaters
Source: Molecules. 2019 Dec 18;25(1):9. doi: 10.3390/molecules25010009 (PMC7017383; doi:10.3390/molecules25010009)
Supplement: Supplementary file 1 [file molecules-25-00009-s001.zip › 3.2.4_gcc-4.4.0.zip!/3.2.4_gcc-4.4.0/3.2.4_gcc-4.4.0/doc/octave/html/interpreter/Formatted-Output.html]

Formatted Output - Untitled


Next: Output Conversion for Matrices,
Previous: Line-Oriented Input,
Up: C-Style I/O Functions


---

#### 14.2.4 Formatted Output

This section describes how to call `printf` and related functions.

The following functions are available for formatted output. They are
modelled after the C language functions of the same name, but they
interpret the format template differently in order to improve the
performance of printing vector and matrix values.

— Built-in Function: **printf** (template, ...)  
> Print optional arguments under the control of the template string
> template to the stream `stdout` and return the number of
> characters printed.
>
>
>
> **See also:** fprintf, sprintf, scanf.

— Built-in Function: **fprintf** (fid, template, ...)  
> This function is just like `printf`, except that the output is
> written to the stream fid instead of `stdout`.
> If fid is omitted, the output is written to `stdout`.
>
>
>
> **See also:** printf, sprintf, fread, fscanf, fopen, fclose.

— Built-in Function: **sprintf** (template, ...)  
> This is like `printf`, except that the output is returned as a
> string. Unlike the C library function, which requires you to provide a
> suitably sized string as an argument, Octave's `sprintf` function
> returns the string, automatically sized to hold all of the items
> converted.
>
>
>
> **See also:** printf, fprintf, sscanf.

The `printf` function can be used to print any number of arguments.
The template string argument you supply in a call provides
information not only about the number of additional arguments, but also
about their types and what style should be used for printing them.

Ordinary characters in the template string are simply written to the
output stream as-is, while conversion specifications introduced by
a % character in the template cause subsequent arguments to be
formatted and written to the output stream. For example,

```
     pct = 37;
     filename = "foo.txt";
     printf ("Processed %d%% of `%s'.\nPlease be patient.\n",
             pct, filename);
```

produces output like

```
     Processed 37% of `foo.txt'.
     Please be patient.
```

This example shows the use of the %d conversion to specify that a
scalar argument should be printed in decimal notation, the %s
conversion to specify printing of a string argument, and the %%
conversion to print a literal % character.

There are also conversions for printing an integer argument as an
unsigned value in octal, decimal, or hexadecimal radix (%o,
%u, or %x, respectively); or as a character value
(%c).

Floating-point numbers can be printed in normal, fixed-point notation
using the %f conversion or in exponential notation using the
%e conversion. The %g conversion uses either %e
or %f format, depending on what is more appropriate for the
magnitude of the particular number.

You can control formatting more precisely by writing modifiers
between the % and the character that indicates which conversion
to apply. These slightly alter the ordinary behavior of the conversion.
For example, most conversion specifications permit you to specify a
minimum field width and a flag indicating whether you want the result
left- or right-justified within the field.

The specific flags and modifiers that are permitted and their
interpretation vary depending on the particular conversion. They're all
described in more detail in the following sections.
